# Ordinary risks and accepted fictions: how contrasting and competing priorities work in risk assessment and mental health care planning

**DOI:** 10.1111/hex.12474

**Published:** 2016-06-17

**Authors:** Michael Coffey, Rachel Cohen, Alison Faulkner, Ben Hannigan, Alan Simpson, Sally Barlow

**Affiliations:** ^1^ Swansea UniversitySwanseaUK; ^2^ LondonUK; ^3^ Cardiff UniversityCardiffUK; ^4^ City UniversityLondonUK

**Keywords:** care coordination, care planning, COCAPP, mental health, recovery, risk

## Abstract

**Background:**

Communication and information sharing are considered crucial to recovery‐focused mental health services. Effective mental health care planning and coordination includes assessment and management of risk and safety.

**Objective:**

Using data from our cross‐national mixed‐method study of care planning and coordination, we examined what patients, family members and workers say about risk assessment and management and explored the contents of care plans.

**Design:**

Thematic analysis of qualitative research interviews (*n* = 117) with patients, family members and workers, across four English and two Welsh National Health Service sites. Care plans were reviewed (*n* = 33) using a structured template.

**Findings:**

Participants have contrasting priorities in relation to risk. Patients see benefit in discussions about risk, but cast the process as a worker priority that may lead to loss of liberty. Relationships with workers are key to family members and patients; however, worker claims of involving people in the care planning process do not extend to risk assessment and management procedures for fear of causing upset. Workers locate risk as coming from the person rather than social or environmental factors, are risk averse and appear to prioritize the procedural aspects of assessment.

**Conclusions:**

Despite limitations, risk assessment is treated as legitimate work by professionals. Risk assessment practice operates as a type of fiction in which poor predictive ability and fear of consequences are accepted in the interests of normative certainty by all parties. As a consequence, risk adverse options are encouraged by workers and patients steered away from opportunities for ordinary risks thereby hindering the mobilization of their strengths and abilities.

## Introduction

Contemporary mental health policy in England and Wales directs that care provision is recovery oriented.^1^, ^2^ The recovery vision requires services to provide the means to enable involvement of people in their care. To do this, people using services need access to information and full involvement in discussions about their care. Care planning in mental health services is a function of systems to enable the co‐ordination and delivery of professionally led intervention. The care plan is the means by which care is articulated and documented. Care plans address a range of mental health needs reflecting the complexity of enduring conditions. These needs include biomedical concerns such as medication effects and psycho‐social aspects such as housing, finances, relationships and daytime activity. Involving people in their care may be unproblematic, but weighty implications arise in more sensitive judgements of risk and safety, particularly where loss of (or restrictions on) liberty can result. A 10% year‐on‐year increase in the use of Mental Health Act detentions in 2014/15 in England has been noted, for example,^3^ alongside excessive use of questionably effective community treatment orders (CTOs).^4^, ^5^ Given the potential for negative outcomes arising from risk assessments, an important question is how contemporary services approach discussions with people about their safety.^6^, ^7^

## Background

Care planning and care co‐ordination has been the primary mechanism for delivery of secondary mental health care in England and Wales for some 25 years.^8^ There has been divergence in policy between the two countries culminating in revisions to the care programme approach (CPA) in England and the introduction of care and treatment planning (CTP) as a legal obligation in Wales.^1^, ^9^ In both countries, providers are required to: comprehensively assess health/social care needs and risks; develop a written care plan (incorporating risk assessments, crisis and contingency plans) in collaboration with the person and their family member or carer(s); allocate a care coordinator; and regularly review care. Care planning is thus seen as a site for the delivery of co‐produced service delivery^10^ within the context of wider involvement practices with individuals^11^ and their families.^12^ Limitations of participation practices^13^ and the potential for pressure applied by workers^14^ however challenge this rhetoric of involvement.

Risk assessment is an important element of care planning and a contemporary concern for workers and service users alike with significant consequences for restrictions on the liberty of patients.^15^ Risk in mental health care is positioned variously across the literature as an on‐going assessment process rather than an outcome,^16^ usually a professional activity and perhaps conversely an outcome arising in social contexts.^17^ In the mental health field, risk is constructed as a potential negative outcome or behaviour arising from the unwanted actions of people using services.^18^ The focus is therefore centred on two main concerns: the risk the person presents to themselves in the form of suicide or vulnerability and the risk the person presents to others. The first of these risks is most common with approximately 5500 suicides each year in the UK, 30% of which are known to mental health services.^19^, ^20^, ^21^

The risk of harm to others is rarer, carries a significant negative outcome for the victim and substantial anxieties for workers and for the mental health system.^22^, ^23^ Risk assessment practices occur within this wider context of concern about possible negative outcomes and uncertain consequences for both the individual being assessed and the assessor. Within the mental health system, there is a contrast between risks perceived to be high profile/low probability (such as dangerous behaviour exhibited by the mentally ill) which call for intervention, and low profile/high probability risks (such as medication effects) that are seemingly accepted without concern.^24^ Judgements about risk therefore highlight certain risks and downplay others and are associated with ‘legitimating moral principles’.^25^ ^(p. 60)^

Risk assessment is a contested area of mental health care. Nevertheless, efforts continue to focus on developing actuarial (meaning measurement) and hence supposedly more scientific mechanisms for identifying and predicting future risk behaviours.^26^, ^27^, ^28^ The predictive accuracy of risk assessment in mental health care is fraught with problems such that even the best actuarial tools perform substantially below that which is commonly acceptable in other branches of healthcare.^23^, ^29^ Reviews have repeatedly noted significant limitations of measurement scales and poor quality assessments with a consistent recommendation that scales are not used for routine clinical practice and calling for a focus on the individual patient.^30^, ^31^

While certain risks take centre stage, more rarely the risks the person themselves are presented with are considered. These risks may be understood as iatrogenic risks, meaning those linked with the provision of care and treatment such as irreversible side‐effects of psychotropic medicine.^32^, ^33^ Risks of discrimination, stigma and possible physical attack have also been highlighted.^34^ People in receipt of services are fearful of losing their independence, of asserting their rights and experience powerlessness in the face of bureaucracy and (sometimes) uncaring staff.^35^ Risks presented by intense scrutiny and follow‐up by workers have also been shown to be a concern for people using forensic mental health services where workers felt compelled to prioritize system concerns of public protection.^22^ Evidence also shows that patients are often unaware of risk assessments taking place,^36^ and that assessments overplay individual factors at the expense of structural, social or interactional issues.^37^ Contrasts in worker and patient assessments highlight that patients soften risk towards others and draw attention instead to vulnerability.^38^ Given the uncertainty around risk decisions, it has been noted that trust is central to engagement and communication between service users and workers.^39^

In this study, we adopt a multiple perspectives approach in which service user, family and worker accounts are obtained to examine the differing views and separate stances of a vulnerable group, their kin and those working with them.^40^ A focus on multiple perspectives may highlight discrepancies between these accounts and signal problems in agreements about treatment goals, risk status and safety.^41^

## Methods

The study protocol for this research has previously been published.^42^ In summary, this was a cross‐national investigation into care planning and coordination across six sites with multiple community mental health teams (CMHTs), designed to explore recovery, personalization and empowerment. Standardized measures were completed by service users (*n* = 449) and care coordinators (*n* = 201); audio‐recorded interviews were conducted using a semi‐structured interview schedule with service providers (*n* = 67), service users (*n* = 33) and family/carers (*n* = 17) (total *n* = 117); and care plans were reviewed using a standard template (*n* = 33).^43^ This study received a favourable opinion from the National Research Ethics Service (NRES) (Ref: 13/YH/0056A).

Anonymized semi‐structured research interview transcriptions and care plan review data related to risk assessment and management were extracted and subjected to an extended in‐depth thematic analysis^44^ to answer the research question, how and in what ways do workers, carers and service users deal with the issue of risk in care planning? The aim was to generate analysis which focused on how participants account for risk status and safety work within the care planning process.

Analysis involved three members of the team independently reading and re‐reading these data, coding and categorizing the material. Emerging categories were shared between members of the team and agreement reached. Using our research question, we interrogated our data to construct themes that focus upon risk discourse as presented by participants. The two themes generated were labelled ‘relationships and involvement’ and ‘the moral work of risk practice’. These themes represent parallel concerns of participants on the one hand to account for the practical everyday concerns of risk and on the other hand the moral work required in risk accounting.

### Findings

Demographic information is presented in Tables 1 and 2. Worker participants included senior managers (*n* = 12), senior practitioners (*n* = 27) and care coordinators (*n* = 28) from a range of professions. The majority were nurses or social workers with more than 10 years of experience in the mental health field and more than 7 years as a care coordinator. Service users (*n* = 33) were predominantly white, two‐thirds were female, in contact with services for more than 10 years with psychosis‐type diagnoses. Family/carer participants (*n* = 17) consisted of more men than women.

**Table 1:** Participant characteristics of community mental health staff^a^

	Senior managers	Senior practitioners	Care coordinators
	(*n* = 12) (%)	(*n* = 27) (%)	(*n* = 28) (%)
Age, years	46 (8) 36–60	45 (7) 32–56	44 (10) 27–62
Gender^1^			
Female	5 (42)	13 (48)	19 (68)
Male	7 (58)	13 (48)	7 (25)
Ethnicity^2^			
White – UK or Irish	10 (83)	17 (63)	19 (68)
White – other European	–	2 (7)	1 (4)
White – other	–	2 (7)	–
Indo‐Caribbean	–	1 (4)	2 (7)
Bangladeshi	1 (8)	1 (4)	–
Indian	–	2 (7)	–
Black African	–	2 (7)	3 (11)
Black Caribbean	–	1 (4)	–
Profession^3^			
Mental Health Nurse	8 (67)	9 (33)	16 (57)
Social worker	2 (17)	6 (22)	6 (21)
Occupational Therapist	–	2 (7)	3 (11)
Psychologist	–	1 (4)	–
Psychiatrist	–	6 (22)	1 (4)
AMHP	1 (8)	2 (7)	–
Other	1 (8)	1 (4)	–
Education^4^			
Doctorate	1 (8)	2 (7)	–
Degree	4 (33)	7 (26)	7 (25)
Masters	3 (25)	7 (26)	6 (21)
Postgraduate Dip/Cert	3 (25)	5 (19)	6 (21)
Diploma/similar	1 (8)	6 (22)	7 (25)
Time working in Mental Health^5^			
10+ years	12 (100)	22 (81)	16 (57)
7–9 years	–	4 (15)	6 (21)
4–6 years	–	1 (4)	2 (7)
1–3 years	–	–	2 (7)
Time as care coordinator^6^: *n* (%)			
10+ years	1 (8)	9 (33)	9 (32)
7–9 years	1 (8)	7 (26)	5 (18)
4–6 years	2 (17)	5 (18)	6 (21)
1–3 years	2 (17)	–	3 (11)
<1 year	–	1 (4)	3 (11)

Dip/Cert, Diploma/Certificate; AMHP, Approved Mental Health Professional.

Missing Data: ^1^Gender, senior practitioner (*N* = 1), care coordinators (*N* = 2); ^2^Ethnicity, senior manager (*N* = 1), care coordinators (*N* = 2); ^3^Profession, care coordinators (*N* = 2); ^4^Education, care coordinators (*N* = 2); ^5^Time working in mental health, care coordinators (*N* = 2); ^6^Time working as a care coordinator, senior managers (*N* = 6), senior practitioners (*N* = 5) and care coordinators (*N* = 2).

a All values represent *n* (%) or mean (standard deviation) and range.

**Table 2:** Participant characteristics of service users and carers^a^

	Service users	Carers
	(*n* = 33) (%)	(*n* = 17) (%)
Age, years	45 (10) 22–65	57 (10) 39–70
Gender		
Female	22 (67)	7 (41)
Male	11 (33)	10 (59)
Ethnicity^1^		
White – UK or Irish	25 (76)	8 (47)
White – other	3 (9)	4 (23)
White other European	–	1 (6)
Bangladeshi	3 (9)	–
Black Caribbean	1 (3)	1 (6)
Indo–Caribbean	–	I (6)
Daytime activity^2^		
Full–time employment	2 (6)	1 (6)
Part–time employment	3 (9)	1 (6)
Education/Training	1 (3)	–
Unemployed	13 (39)	4 (23)
Voluntary work	4 (12)	1 (6)
Other	10 (30)	9 (53)
Time in mental health services^3^		
10+ years	20 (61)	10 (59)
7–9 years	5 (15)	3 (18)
4–6 years	3 (9)	–
1–3 years	3 (9)	1 (6)
<1 year	1 (3)	3 (18)
Relationship status^4^		
Single	18 (54)	5 (29)
In established relationship	13 (39)	12 (71)
Mental health problem^5^		
Psychosis/Schizophrenia/Bipolar Disorder	9 (27)	–
Psychosis and substance use	1 (3)	–
Psychosis and depression	6 (18)	–
Psychosis, depression and substance use	3 (9)	–
Psychosis and other	1 (3)	–
Depression/Anxiety	7 (21)	–
Other	3 (9)	–
Frequency of contact with care coordinator^6^		
Daily	3 (9)	**–**
Weekly	12 (36)	–
Monthly	8 (24)	–
Other	8 (24)	–
Not applicable	1 (3)	–
Frequency of contact with carer^7^		
Daily	23 (70)	–
Weekly	2 (6)	–
Monthly	1 (3)	–
Other	6 (18)	–

Missing Data: ^1^Ethnicity, service user (*N* = 1), carer (*N* = 1); ^2^Daytime activity, carer (*N* = 1); ^3^Time in mental health services, service user, (*N* = 1); ^4^relationship status, service users (*N* = 2); ^5^Mental health problem, service user (*N* = 1); ^6^Frequency of contact with care coordinator was not collected from one service user, and ^7^Frequency of contact with carer was not collected from one service user.

a All values represent *n* (%) or mean (standard deviation) and range.

### Relationships and involvement

In the following extracts, service user participants from contrasting rural and urban areas in England indicate the potential negative effects of poorly established relationships with workers.

…since I came out [of hospital]…I wasn't feeling safe, and cut an artery and ended up in theatre. And after that I didn't get any extra support… I don't know them well enough to sit and talk to them.…because I don't know if I'm going to see that person again … so I don't want to open up to them. (Service User)

…. the less you hear from [your care coordinator] the more distance you feel about the relationship and it becomes difficult to ask them for help. … I felt really suicidal about three or four days ago… because I thought, I can't go to these people. It's terrible that I have a team around me that I won't approach.… Do you know there's a million ways you can contact me. It only takes one, a text, a phone call, an email, anything, a letter. (Service User)

Relationships enable or inhibit communication of safety concerns.^45^ Stability in these relationships appears to be vital when discussing distressing and worrying experiences. In the absence of stable relationships with workers, service users can feel isolated from help and left to manage their safety alone. Fewer service user participants felt engaged and supported to consider their safety as in the following abstract from an urban site in Wales.

[risk has been discussed and considered]… because when I went out they were concerned about how I would cope and how I would deal with things and contingency plans… we had contingencies in place for things going wrong and that I would be safe no matter what because I wouldn't be on my own and we'd all discussed how things would be dealt with if there was a problem. (Service User)

Involving the person can also provide opportunities to make use of wider support networks so that safety and risks of relapse are included as indicated by this participant from another English rural site.

Yeah, risk [has been discussed with me] on several occasions… [and with] CPN [community psychiatric nurse], even my family and friends… if I want to confide in someone, they know certain risks, risk factors and other things that could cause relapses. (Service User)

Participants in our study and elsewhere^46^ present risk as a worker priority. For example, in the following extracts, service user participants from sites in England and Wales indicate that they see care planning and safety as tasks that workers must do for the purposes of deflecting claims of responsibility rather than designed solely with their interests at heart.

[safety and risk] was their conversation, not my conversation. Risk and safety and what have you, they perceived it wrongly. (Service User)

this is why I say that the care plan is for the professionals because the care plan is about protecting them from culpability I think which is why safety is so prominent in it… (Service User)

Workers acknowledge they do not involve service users in risk assessment discussions, and some accept that their practice is conservative and errs on the side of caution. For example, this data extract from a senior manager in one CMHT in Wales gives an indication of a wider cross‐national pattern.

whilst they may be engaged in the care plan, and that's debatable, with risk assessment, it's, that's one thing we never, you never discuss with service users just in case it alarms them. (Senior Manager)

Workers acknowledge too that concerns about risk in care plans may not be shared because of the potential for disagreement about the focus of these plans. An example is shown in the data extract below from a care co‐ordinator in another Wales CMHT site.

There have been case reviews and … it does kind of raise your anxieties and you may feel that certain things may need to be put into people's care plans, where the client wouldn't necessarily agree with that, so wouldn't feel that they would share the same concerns about risk as you would. (Care Co‐ordinator)

Service user and family participants indicate that workers do not discuss risk with them. For example, the following data extracts are from two different English NHS sites and describe a common pattern across all six sites in our study.

nobody has spoken to me at all… I don't think she's been asked in [research site], since we've been here, whether she is a threat to herself, whether she is a danger to herself in anyway. (Family member)

[has risk/safety been discussed with you?] No… I haven't noticed it. (Family member)

This seems to deny service users opportunities to engage and be involved in discussions about their safety and the consequences arising from risk assessments. Involvement in decisions about one's care is seen as central to health policy approaches so that individuals have more say and are better informed.^47^ It is not clear from our data that all patients see themselves as active health consumers. Workers are ambivalent about the possibilities of involvement tending to emphasize possible negative outcomes and as this senior practitioner in one CMHT in Wales intimates not discussing risk with those involved may be well intentioned, but also something that workers claim is embarrassing.

To my shame, there are cases that I follow that culture, that I hide that risk assessment or secret. Why? Because I want to protect the individual from the knowledge of that.., their illness that they have can be a risk to themselves or to the others. It's a practice that I'm not very comfortable but nevertheless, I raise my hand and say I have. (Senior Practitioner)

Although some service users report being involved in risk discussions, for the most part, they position themselves as passive recipients of instructions given by workers rather than participating in decisions on a shared basis.

Family members too have limited input to risk assessment and management plans to the extent that they report a sense of being unsupported and left to manage situations of increased risk. For example, the data extract below is taken from a research interview with a family member in one English NHS site who felt that they had been left to manage safety and risk themselves.

I: Do you feel your safety and the safety of [participant's name] have been considered in their care planning and coordination?

P: No, definitely not, 100% no way. I've stopped her cutting herself loads of times, I've stopped her taking overdoses, I've had to hide tablets, all sorts of stuff… nothing's been discussed with me, no. (Family member)

There are contrasting accounts from participants of their experiences of risk assessment practices. In the data extract above, we see one carer expressing something close to exasperation that whatever risk assessment and management practice is operating, it is largely unknown to the person and not managing risk behaviours. Service users and their families are placed in an invidious position. On the one hand, they are positioned passively by being excluded from involvement in risk discussions, which works to deny them agency. On the other hand, patients and their families feel they are responsible for chasing up their own support or initiating contact in the event of a crisis event, suggesting that in order to get help they have to be active and agentive.^48^

The picture is not universally negative on involvement, but we calculate that as many as four times more people reported not being involved in their risk assessments than those providing more positive accounts of involvement. This was further substantiated when we looked at actual care plans across our six sites. From a total of 33 care plans reviewed for this study, we found 12 showing individuals’ views were considered in risk assessments. However, four of these 12 did not show evidence of extending this to the risk management plan. Twenty‐one care plans showed risk assessments that did not involve the views of the person.

In summary, discussions about risk and decisions arising from these assessments rarely involve the person. In many cases, where care coordinators say they involve people, this appeared to be for the purposes of answering assessment questions only. It is not clear that the purpose of these assessments is ever made known to service users, and workers indicate this is to prevent upset or alarm to patients.

In the next section, we develop our analysis to examine the moral work of risk practice as displayed by participants and the purposes this is put to in discussions about risk, blame and concerns about achieving a balance between individual autonomy and alternative practices that work to limit autonomy.

### The moral work of risk practice

The moral work of risk practice features in the talk of participants across all groups in this study. Moral work refers to how participants use accounts to position activities in relation to ascribed positive or negative values. Our use of moral work is derived from earlier work on moral tales in which speakers have been shown to ‘attend to the issue of their appearance as moral persons, competent members and adequate performers’ and in so doing “accomplish the status of moral adequacy”.^49^ ^(p. 276)^ Moral work appears to be required in situations of doubt and uncertainty where the rationality of individuals themselves is open to question. For example, risk practice which is complex and difficult may be constructed by workers as high value especially given the uncertainty of the possible outcomes. Moral work is achieved by care coordinators in their displays of professional judgement and positioning of their decisions as reasoned with the best interests of the person in mind. Intervention by workers in the lives of people using mental health services is legitimated by concerns about risky behaviours and their prevention. The moral stance in this sense is taken to be socially derived, contingent and determined in interaction with others.

For the person and their family, there is significant weight attached to determinations of risk in the form of losing their liberty and being separated from loved ones for extended periods of time. The conclusion that someone is unsafe places them in a morally ambiguous position and opens the way for the application of value‐laden labels that are difficult to shake off. Being seen to be unsafe denotes a more general sense of riskiness that could be taken to imply someone is also a danger to others. Family members, as shown in this data extract from one of our Welsh sites, work to establish the moral standing of the person in the absence of an explicit category.

she wasn't a risk to others. And they basically talked to her and realised that it was just a passing fancy, it was now over, and they didn't worry too much about it, so (Family member)

Family members place the emphasis as being centred on risk to self rather than risk to others, but also something temporary and transient. Risk is therefore not to be seen as permanently implicating the character or identity of the person. ‘A passing fancy’ works here for this purpose, and for emphasis, the speaker indicates that professionals are not overly concerned themselves. Service user accounts do moral work in attending to what might be regarded as the classic sick role requirements, for example seeking help and following professional advice.^50^ Another feature of the accounts of service user participants seen in this data extract from one of our English CMHT sites is that they work to highlight that the danger is to themselves rather than others.

I've never had anyone that can understand the safety towards myself. Through the whole of my illness they've been more worried about safety to other people, and I would never have hurt anybody, in any shape or form, than they were about safety towards me. And I was a danger to me. (Service User)

It has been noted that moral work in accounts operates as a form of biographical repair,^51^ and here, the participant engages in this repair by managing their moral identity as an ethical subject. This is a means to negotiate the tricky terrain of negative evaluations of being risky and mentally ill and for workers and families perhaps too the avoidance of stigma by association.^52^

Workers speak of risk assessment practices carrying a sense of moral ambivalence. They see a tension between the rhetoric of recovery and the negative, and potentially restrictive, outcomes of risk assessment practices. A senior practitioner in one of our Welsh CMHT sites summed this as follows:

the stigma of the mental health is still very prevalent in our society so by doing a risk assessment you more or less emphasize that stigma … You are a very risky person, you're dangerous to yourself, and you're dangerous to society, whereas this doesn't go well with the recovery that we try to achieve for that person. (Senior Practitioner)

It has been argued that risk management practice is discriminatory given the disproportionate attention and calls for compulsion and control directed towards those with mental ill health.^53^ Risk assessments are laden with the implications of their outcomes and concerns about failures to predict what may be unpredictable and consequent apportioning of blame.^54^

## Discussion

### Ordinary risks and accepted fictions

Our study shows that risk is a significant concern for workers, but is rarely discussed openly with service users. This limits both the potential for greater involvement by individuals and families in exploring and managing safety and the potential for positive risk‐taking to become an integral part of their recovery. Positive risk‐taking involves the weighing up of autonomous decision making to determine the ‘potential benefits from exercising one choice of action over another’.^55^ ^(p. 5)^ People using mental health services clearly wish to be safe, but are nonetheless aware that procedures ostensibly designed to enable this safety may work in ways which limit their opportunities for establishing recovery and continued liberty. Care coordinators also recognize that risk assessment and management practices may work in opposition to the goals of mental health recovery.

Risk assessment and management practices operate in ways that suggest the use of ‘accepted fictions’ about these practices. For our purposes ‘accepted fictions’ are those stories that workers, families and service users produce or rehearse to facilitate day‐to‐day work of mental health care. The concept is derived from the notion of ‘legal fictions’ which Bernat notes is a social construct for the purposes of legally defining ambiguous situations:^56^ for example, for the purposes of determining whether someone is legally considered, for all intents and purposes, to be blind. For our purposes, legal definitions of risk are not required; however, the notion of accepted fictions recognizes that risk status is ambiguous, outcomes uncertain and consequences significant. Accepted fictions therefore centre on the ambiguity of risk assessment practices that are either transparently ineffective or for which the contested knowledge about them is known, but largely ignored. These fictions appear to operate to legitimate practices that cause moral unease. In this sense, all parties in an interaction may be aware that a proposed approach is known to be largely for administrative purposes and has little or no scientific validity. Risk assessment is positioned as objective by workers often in the absence of scientific verification and despite the limited evidence for its predictive ability.^29^ Workers are nevertheless compelled to demonstrate that risk has been considered and that safety is being monitored. The ability to conduct assessments and monitor risk is a matter of professional competence. As a result, workers are concerned with demonstrating that risk assessment has taken place to their colleagues, if not to the patient and their families. In our interviews, they rarely if ever explicitly question the practice, its efficacy or the purposes it serves. These fictions take various forms and extend to the claim that discussing risk and involving people will cause upset, worsen the patient's condition and hinder alliances. The conclusion that can be drawn is that this fiction largely operates as an explanatory device for workers who find (or imagine that) such conversations (are) difficult. Risk language has been noted to be largely negative and inclined towards unpleasant outcomes; hence, these fictions may work to preserve working relationships which would otherwise be challenged by a focus on assessments that have limited value in themselves.^26^

Workers treat risk assessment as a separate function within the care planning process despite its central role in care coordination in England and Wales. By separating out risk from usual care planning activities, care coordinators appear to prioritize the protection of the working alliance over helping individuals learn about potential risks. One consequence is that people using services and their family members are not fully involved in the process of risk assessment and remain uncertain if plans are in place to deal with safety concerns. Family members appear to be reassured that some risk assessment has taken place, although for the most part they take this on trust. They want clearly laid out plans detailing who to contact in an emergency and a prompt response from services in such circumstances.

Involvement requires overcoming some significant hurdles as workers appear wedded to an overly paternalistic view of individuals and their presumed risk status. For example, involvement was often positioned as an aspiration to be achieved rather than something that was commonly practised and accompanied by the caveat ‘if appropriate’. Moral work is required of professionals labouring under their own legitimacy crises, although longer term redemption may only be achieved by fully engaging with involvement practices.^57^

Some service user participants suggested benefits in discussions on risk and its management. These data raise the possibility that where agency or autonomy is honoured, there is room for the active health consumer in contemporary constructions of mental health risk assessment. The contrast between the managed patient and the ideal patient as an autonomous, reflexive and active consumer of health care creates a tension for mental health services and those they serve. Neoliberal discourses position the ideal health consumer as someone who takes responsibility for the maintenance of their own health rather than depending on professionals for this.^58^ Workers and patients may however have alternative and competing normative versions of what it means to be a mental health patient.^59^ For both parties, the presence of mental health legislation features as an important backcloth, although this plays out very differently for each group. Power lies with workers who may deploy statutory powers depending on determinations of risk status. For patients, the ever‐present threat is that they may lose their liberty and be compelled to accept treatments that they would not otherwise choose. The contrast with notions of the ideal health consumer is that in mental health settings, passive subjects are background expectancies for workers. Agency is denied either overtly in the use of mental health legislation to restrict liberty and impose treatments or covertly in the application of risk plans that limit or curtail individual choice. Our data indicates that workers believe service users do not want to be involved in critical decisions which ultimately determine opportunities to move towards greater autonomy. This is a form of epistemic injustice that denies patients opportunities to develop knowledge about their experiences.^60^ It withholds key information on conclusions about risk status on which consequential decisions will be made such that they are then unable to rectify or retrieve their situation.

An abiding conundrum of contemporary mental health services is that neoliberal attempts to construct the prudent patient as a responsible and active participant in their own care also positions the patient as culpable and blameworthy. Mental health service users are expected to learn to manage their own care and recover whilst simultaneously being the focus of suspicion and doubt in relation to their risk status.

Risk assessment practice informs the use of community treatment orders after discharge from hospital^5^ so that a failure to involve people in these processes places them at a significant risk of continued restrictions on their liberty. Risk is therefore transformed from a concept into a process which is then itself applied to aid decisions on how specific sets of situations should or ought to be managed. In some cases, this can work to the benefit of the individual, their families and wider society. For example, temporary detention and treatment as an outcome has been accepted by some service users as being in their own best interests as long as there is a sense of procedural justice.^61^ Risk assessments carry significant weight in the present. Those subject to them have limited input to determinations and little sense of procedural justice from the assessment process. Once a person is given the label of ‘risky’, they may struggle to remove it. There is evidence that such assessments can be inaccurate with historical information being used for decisions in the present.^62^ Workers erring on the side of caution can thus deprive people of opportunities to move on, try new ways of living and recover.^63^

An alternative to current practice is to develop care coordination so that individuals benefit from social bonding, adjustment and integration with the aim of sustaining community living. Direct involvement of people in their own risk assessments may lead to more well‐informed assessments and open up the possibility of focusing on micro‐level relationships (individuals, family, household, community) that enable people to benefit from supports that in themselves can successfully manage or reduce risk behaviours and aid recovery.^64^, ^65^ Care coordinators could engage in conversations about risk with people they work with allowing service user and professional accounts to stand side by side as credible versions of the day‐to‐day realities of living with mental distress. This will not only allow service users to benefit from the expert opinion of care coordinators, but help workers to see the broader range of risk concerns that people encounter in their everyday lives.

## Conclusion

A surprising finding of our study is that objectives of recovery including self‐management, self‐determination and responsibility are not extended to risk practices. Previous research has noted that people were not involved or aware of assessment of risk behaviours towards others^36^ and that potential exists for directly engaging people in their own risk assessment and management.^66^ The current study adds new analysis highlighting that workers, families and service users are moved to provide accounts which do moral work in situations of ambiguity and moral unease. The notion of accepted fictions in situations of uncertainty and ambiguity provides one explanation for practices and may provide a form of normative certainty to salve the moral unease of actors.

Genuine involvement of service users in risk decisions is perhaps then a key marker of whether services are truly recovery‐focused. Care planning and its associated risk assessment and management plans may operate for workers, families and patients alike as forms of ‘accepted fictions’, as stories told to assure the system and each other that risk is being monitored and that everyone will be safe.

## Funding

Health Services and Delivery Research Programme, (11/2004/12). The views and opinions expressed herein are those of the authors and do not necessarily reflect those of the HS & DR Programme, NIHR, the National Health Service (NHS) or the Department of Health.
